# Exploring the mediation role of hardiness in the relationship between feedback sensitivity and test anxiety among nursing students: a multi-site inquiry

**DOI:** 10.1186/s12912-025-02946-9

**Published:** 2025-04-15

**Authors:** Alaa Eldin Moustafa Hamed, Mohga Fathy Hamza, Nahla Abdallah Abd El-Tawab, Ahmed Abdellah Othman, Abeer Moustafa Barakat, Mohamed Hussein Ramadan Atta

**Affiliations:** 1 Present Address: Psychiatric/Mental Health Nursing Faculty of Nursing, Helwan University, Cairo, Egypt; 2 Psychiatric and Mental Health Nursing, Faculty of Nursing, Cairo University, Cairo, Egypt; 3 Nursing Administration, Faculty of Nursing, Sohag University, Sohag, Egypt; 4 Maternal and Newborn Health Nursing, Faculty of Nursing, Helwan University, Cairo, Egypt; 5 Nursing Department College of Applied Medical Sciences, Prince Sattam Bin Abdulaziz University, Wadi Addawasir, Saudi Arabia; 6 Psychiatric/Mental Health Nursing, Faculty of Nursing, Alexandria University, Alexandria, Egypt

**Keywords:** Feedback sensitivity, Hardiness, Test anxiety, Nursing students

## Abstract

**Background:**

Academic students often face significant academic pressures that can lead to test anxiety, affecting performance and well-being. Students who are highly sensitive to feedback may experience heightened levels of anxiety due to their perceived ability to meet expectations and their reaction to criticism or praise.

**Aim:**

This study assessed how hardiness moderates the relationship between feedback sensitivity and test anxiety among nursing students.

**Methods:**

A multicenter descriptive correlational design was utilized, involving a convenient sample of 1932 Egyptian nursing students. Data were collected conveniently and analyzed to determine the correlations between test anxiety, hardness, and feedback sensitivity scale from January 2024 to February 2024.

**Results:**

The study revealed that 52.9% of the nursing students experienced a high level of test anxiety. A statistically significant positive correlation was found between feedback sensitivity and test anxiety (*P* < 0.001), while a negative correlation was observed between hardiness, feedback sensitivity, and test anxiety (*P* < 0.001). Predictors of test anxiety, based on the linear regression model, included gender, educational level, monthly income, previous grades, history of failure, use of non-prescribed sedatives, hardiness, and feedback sensitivity (*P* < 0.01 or *P* < 0.001). Mediation analysis indicated that feedback sensitivity had a direct effect on test anxiety (*P* < 0.001), as well as an indirect effect through hardiness, which played a mediating role in the relationship between feedback sensitivity and test anxiety (*P* < 0.001).

**Conclusion:**

This research underscores the critical role of feedback sensitivity and hardiness in shaping test anxiety among nursing students.

## Introduction

Mental health affects students’ stress management ability, learning engagement, and effective performance in both academic and clinical settings. Mental well-being is crucial for student success and retention in nursing programs and professional competence growth [1]. Nursing education is inherently stressful due to rigorous coursework, clinical rotations, and the emotional demands of patient care. Adequate support systems, including counselling services, peer support, and faculty mentorship, significantly promote mental health [2].

Academic performance reflects the acquisition of essential knowledge; successful academic performance is necessary for passing licensure and certification exams, which can produce anxiety related to these exams [3]. Test anxiety is a specific form of performance anxiety characterized by excessive worry, fear, and apprehension about tests or exams. It can significantly affect the student’s performance at school and personal life [4, 5]. Test anxiety is not simply a case whereby a student gets jittery before a test. However, it is a level of stress that might disrupt one’s concentration, memory, and other test performances [6]. Nist and Diehl 1990 conceptualized test anxiety as a range of emotional, psychological, and behavioral reactions that occur when individuals worry about the potential adverse outcomes or failure in exams or similar evaluative situations [7].

Studies show that nursing students face test anxiety more often than students in other disciplines because of the pressure to achieve both academic and clinical competencies, as well as the high-stakes nature of their exams [8]. It was reported that nursing students who participated in their study experienced high levels of test anxiety, with 30–50% [9] and 33–50% prevalence [10]. Moreover, A study performed by [11] denoted that approximately 44% of nursing students faced moderate to high levels of test anxiety.

Test anxiety significantly impacts the academic performance of nursing students; this leads to lower grade achievement and a higher risk of educational failure and causes avoidance behaviors, reduced study time, and ineffective study strategies [12]. Poor academic performance results from test anxiety and can create a vicious cycle and be malicious to the student’s academic journey and overall educational experience [13]. Besides that, chronic test anxiety leads to mental health problems, including generalized anxiety disorder, depression, and burnout [14]. Persistent anxiety negatively influences students’ mental health and well-being, reducing their ability to cope with academic and personal life challenges [15]. In addition, test anxiety is associated with a range of physical health problems, such as headaches, gastrointestinal issues, and weakened immune function. These physical symptoms can further impact students’ ability to perform academically and maintain a balanced life [16]. The combination of mental and physical health issues associated with test anxiety can significantly diminish the quality of life for nursing students. It affects their relationships, reduces enjoyment of their educational experience, and undermines their overall well-being [17].

Feedback sensitivity is the extent to which students are most affected by feedback, predominantly negative or critical feedback. Students with high feedback sensitivity usually tend to have a stronger emotional response to such feedback, which affects their self-concept, motivation, and behavior [18]. Constructive and positive feedback boosts motivation, supports learning, and fosters persistence. In that respect, students who are highly sensitive to feedback may enjoy enhanced self-efficacy and willingness to learn. However, the inclusion of negative feedback could have the effect of lessening interest in students, especially those sensitive to feedback. Therefore, criticism may be perceived as devaluing general abilities rather than specific approaches needing improvement, reducing motivation and engagement among students with high feedback sensitivity [19].

Highly sensitive students may experience reduced self-esteem following negative feedback, which can affect their overall academic performance and willingness to take risks in learning [20]. Students with high feedback sensitivity might develop a negative self-perception if they frequently receive critical feedback. This will make them feel inadequate and condition their minds so that their abilities are fixed and cannot be changed. High sensitivity to feedback can result in performance anxiety due to the risk of negative evaluation and subsequent emotional penalties. Such anxiety will eventually hinder performance skills during assessments and active involvement in class [21]. Besides, how students process and react to feedback determines the kind of learning outcome they realize. Constructive feedback is essential for identifying improvement areas and fostering a growth mindset. However, highly sensitive students might struggle to view and use feedback to enhance their learning objectively [22].

Hardiness or psychological resilience is commonly understood as a worldview or attitude that forms early in life and is generally stable over time. However, it can be modified in specific situations. Hardiness is a personality trait that involves a resilient attitude towards stress and adversity. It is characterized by feelings of control over life events, commitment to participating in life activities, and viewing challenges in life as an opportunity for growth [23]. Hardiness is very important since it protects one from stressors, leading to improved mental and physical health. In addition, hardiness allows students to deal more effectively with stressors, reducing the negative effect of stress on well-being. It mitigates test anxiety by building effective coping strategies and enhancing resilience, leading to a positive attitude toward challenges [24].

Hardiness encompasses three components. The first is commitment, which refers to an individual’s tendency to engage with and involve themselves in various activities and roles. It signifies a sense of purpose and meaning in one’s actions [25]. Control is the belief that one can influence the events and outcomes in one’s life. Hardiness extends to include a sense of autonomy and self-efficacy [26] and Challenge that involves perceiving potential stressors experienced by other people as opportunities for growth and development rather than threats rather than as threats reflect an optimistic and proactive attitude toward change. Viewing challenges positively enables fast adjustment to new situations and quick recovery from setbacks. It brings out a growth mindset whereby a person learns from their experiences and develops from them [27].

This increased emotional arousal and cognitive interference can reduce self-esteem, lower self-efficacy, and increase avoidant behaviors, thereby increasing anxiety and poor test results. In this manner, students may find themselves trapped in a cycle where their sensitivity to feedback perpetuates their anxiety, further diminishing their academic performance and overall well-being [28]. Hardiness plays a critical role in allowing students to build up a robust coping mechanism that can, in turn, moderate or diminish the adverse effects of stressful events associated with academic life. Commitment, control, and challenge dimensions of hardiness stimulate students to stay engaged in their learning, exercise control over the same through meaningful efforts, and perceive challenges as opportunities for personal and academic growth. These attitudes engender resilience, enabling the student to cope and ‘bounce back’ from setbacks, thereby maintaining mental health in the face of academic pressures [27]. Hardiness builds stress management capabilities, reduces test anxiety, and creates a much more positive educational experience, thus sustaining academic success and psychological well-being [29].

The transactional Model of Stress and Coping of Lazarus and Folkman denotes a framework for understanding how individuals perceive and explain that stress is not a response to external events but a dynamic interaction between the individual and the environment [30]. In addition, Kobasa’s hardiness theory regards hardiness as a personality attribute that buffers one from stress and creates possibilities for adaptive coping [31, 32]. Hardiness encourages adaptive ways of coping with and managing stress through commitment, control, and challenge [33]. This hardiness factor not only acts against the harmful effects of stress but also helps promote general well-being and performance in all spheres of life. Kobasa’s theory highlights the need to develop hardiness to increase resilience against stress and promote healthy, adaptive responses to the demands of life [34].

Findings of the previous research have disclosed that feedback sensitivity and hardiness, as personality variables, have an independent effect on test anxiety levels in nursing students. However, there is a notable lack of comprehensive studies examining their combined effects [35]. Existing literature primarily discussed every one of these factors in isolation, leaving a wide-open significant gap in understanding how their interaction influences test anxiety. This gap adds more weight to the need for further research on how high feedback sensitivity and other factors, such as varying levels of hardiness, are related to anxiety in nursing students during exams [36]. To fill this gap, much research will be required to integrate both psychological and educational variables, providing a holistic understanding of test anxiety. Complementing this with insights from other disciplines could give a dimension of complexity that different influences have on the level of anxiety students experience. In this regard, an integrated approach can help identify specific stressors and coping mechanisms, thus creating an all-inclusive framework to understand and deal with test anxiety in nursing education [37].

It is essential to fill this research gap because targeted interventions and support mechanisms for nursing students are at stake. This will not only manage and reduce anxiety but also enhance the student’s academic performance and support their well-being overall, leading to a more effective and resilient nursing workforce [38]. Since nursing students will be the future workforce in the area of medical science, the study of feedback sensitivity, hardiness, and test anxiety among nursing students is critical in improving educational outcomes, mental health, and well-being; increasing the rate of retention; developing competent health professionals; and supporting research and policy development. These factors play a significant role in shaping the experiences and success of nursing students, ultimately influencing the quality of care they provide in their professional careers.

## Methods

### Aim of the study

This study explored the mediation role of hardiness in the relationship between feedback sensitivity and test anxiety among nursing students.

### Setting and study design

The study was conducted in nursing educational institutions, including faculties of nursing (both governmental and private) and technical nursing institutes affiliated with the Ministry of Higher Education and the Ministry of Health, across Upper Egypt, Eastern Egypt, Western Egypt, the Delta area, and the Center. A descriptive correlational design was utilized to carry out the study.

### Participants

During their examination period, a convenience sample of 1,932 Egyptian nursing students from various regions of the country was employed. This diverse sample comprehensively represents the student population across different geographic areas and academic contexts [39, 40].

### Sample size and study sampling

The sample size was determined using a sample size equation, considering a significance level of 5% and a study power of 80%, based on data from existing literature. The sample size was calculated using the following formula:

$$\:\text{n}\:=\frac{{\left(\text{Z}1-\frac{{\upalpha\:}}{2}\right)}^{2}.{\text{S}\text{D}}^{2}}{{\text{d}}^{2}}$$

Where Z1-α/2 = is the critical value for a 95% confidence level (1.96), SD is the standard deviation of test anxiety, and d is the desired absolute error or precision. Based on this calculation, the required sample size was 1932 participants.

The study included a convenient sample of 1932 Egyptian undergraduate nursing students from different nursing institutions, aged 18–30 years. Participants were selected from accredited nursing programs and excluded if they had a comorbid anxiety disorder (verified through health insurance records). Expatriate non-Egyptian students were also excluded to avoid the confounding effect of alienation-related anxiety. Data was collected during examination periods to assess the real-time impact of feedback sensitivity and hardiness on test anxiety.

### Study instruments

Four validated instruments were employed to gather data for this study, including participants’ basic and educational information, a Short Hardiness Scale, a Test Anxiety Questionnaire (TAQ), and an Instructional Feedback Orientation Scale (IFOS). These instruments were distributed in an online self-administered form in the participants’ mother tongue (Arabic). The researchers translated the scales initially developed in English into Arabic using the committee approach to translation [41]. The committee included four members who translated the scales independently and in parallel. Among these four were two nursing professors from Egypt, a nurse proficient in English and experienced in healthcare settings, and an English educator native and fluent in English. Every committee member accepted the final versions of each scale. Microsoft Forms application was used to create this anonymous survey tool. A panel of experts in psychology, psychiatry, and psychiatric nursing then evaluated the instrument’s validity and suitability for use with nursing students. To accommodate the local customs of Egyptian students, the experts also modified and added several questions. A pilot study involving 190 participants further confirmed the questionnaire’s validity. Then, the researchers distributed the questionnaire to staff members at the different educational nursing institutions.

This research instrument encompasses four primary sections, the first one containing the participant’s basic and educational information, including age, gender, level of education, place of residence, marital status, type of education, academic grade, history of academic difficulties, and the possibility of using non-prescribed sedatives during exams.

The second section was the Short Hardiness Scale, developed by Bartone [40, 42]. SHS is a self-report of 15 positively and negatively keyed items that assess the three proposed domains of hardiness: commitment to life’s tasks, control over life’s outcomes, and perception of challenge in dealing with ambiguous life events. The scale used for measuring psychological hardiness encompasses 15 items. Scoring criteria: As for the scoring system of SHS, responses of items were against a 4-point Likert scale with anchors 0 (not at all true) to 3 (completely true). To obtain scale and subscale scores, sum responses to items and appropriate subscale items. CM = commitment (1,4*,7,10,13*); CO = control (2,6,8*,12,15); CH = challenge (3*,5,9,11*,14*). Total hardiness = Sum of (CM + CO + CH). Cronbach’s alpha coefficient for the total hardiness measure is 0.83, and for the facets (subscales), 0.77 (commitment), 0.71 (control), and 0.70 (Challenge).

The third part of the research instrument was the Test Anxiety Questionnaire (TAQ), a 10-item self-reporting scale developed by Nist and Diehl (1990) [43]. This instrument determines if a student experiences mild or severe test anxiety, with a 5-point rating scale ranging from (1 = Never) to (5 = Always). As for the Scoring system, scores will range from 10 to 50. A low score (10–19 points) indicates that the student does not suffer from test anxiety. If the score is extremely low (close to 10), more anxiety may be healthy to keep students focused during exams. Scores between 20 and 35 indicate that, although the student exhibits some of the test anxiety characteristics, the stress and tension levels are probably healthy. Scores over 35 suggest that the participant is experiencing an unhealthy level of test anxiety. We evaluated the internal consistency of the questionnaire with Cronbach’s alpha, resulting in a satisfactory value of 0.83.

The fourth part of the research instrument was the Instructional Feedback Orientation Scale (IFOS) developed by King and colleagues (2009) [44]. It is 33 a self-reporting instrument that asks participants to report on their predispositions toward receiving instructional feedback across four dimensions: feedback utility, feedback sensitivity, feedback confidentiality, and feedback retention. Responses were solicited using a 5-point Likert scale ranging from 1 (strongly disagree) to 5 (strongly agree). With scoring ranging from (33–165). Estimates of internal reliability for the IFOS produced acceptable Cronbach’s alpha coefficients for each dimension, ranging from 0.69 for feedback retention and 0.74 for feedback confidentiality to 0.86 and 0.88 for feedback sensitivity and utility, respectively.

### Procedures

The data collection for this study was instituted after obtaining ethical approval from the faculty of Nursing at Sohag University, followed by the approval of the educational administration to which those nursing educational institutions under study belong. The researchers prepared the research instruments after reviewing related articles and periodicals. The instrument was translated from English to the participant’s mother tongue, “Arabic,” at the Center for Specialized Languages of the Faculty of Arts, Helwan University. A panel of experts in psychology, Psychiatry, and psychiatric/ mental health nursing then evaluated the instrument’s validity and suitability for use with nursing students.

The survey was successfully distributed to participants in nursing educational institutions through staff members. The research questionnaire was created using Microsoft Forms and distributed to the participants by the staff members as an initial invitation with a QR code or online link to the survey. The survey contained two parts, with the first part acting as the informed consent, explaining the study’s aim, confidentiality guarantees, and the ability to withdraw from the study at any time without any fear (risks). As a result, informed consent was included in one document with the research tool. Participants had to electronically agree to the terms before continuing with the questionnaire, ensuring they fully understood before proceeding. The second part was the research instrument tools.

**Fig. 1:**
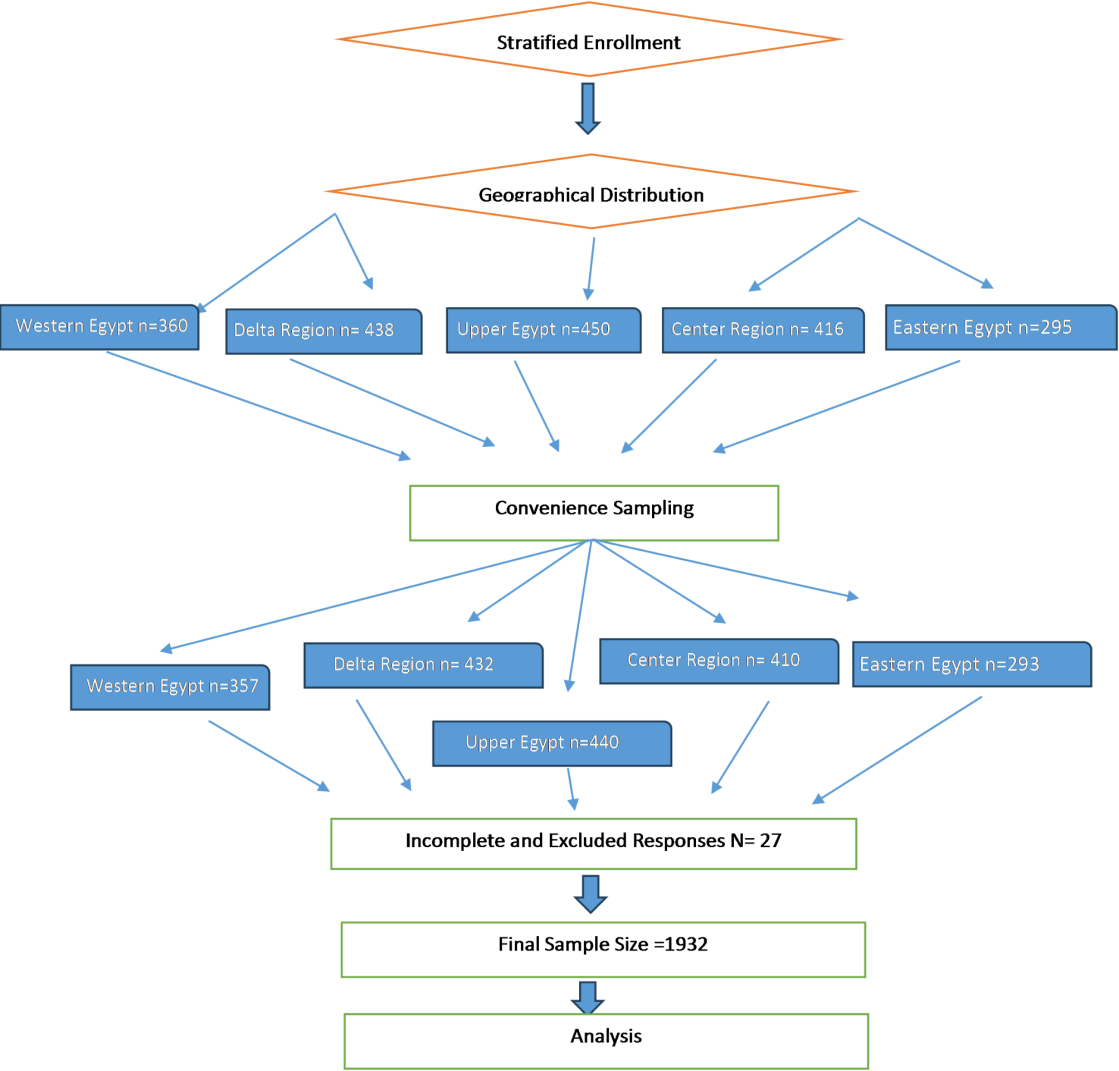
Study sample graph

The study utilized a stratified sampling approach to ensure a comprehensive representation of nursing students from various educational institutions across Egypt. The country was divided into five principal regions: Region 1 (The Center Region), Region 2 (Upper Egypt), Region 3 (Western Region), Region 4 (Eastern Region), and Region 5 (Delta Region). Within each region, nursing students were recruited from educational institutions that represented the diversity of the nursing education landscape. Participants accessed the research instrument by scanning the QR code or using the provided online hyperlink. Data collection timing was during the exams. Responses were automatically collected and stored in Excel, ensuring participant anonymity throughout the survey conducted via WhatsApp. All study procedures were conducted within the ethical guidelines outlined in the Declaration of Helsinki and its later amendments. Figure (1) shows the study sample graph.

### Statistical analysis

Data analysis was performed using SPSS 26.0 (IBM et al., USA) to examine the survey responses from the recruited students. Additionally, all variables underwent a Shapiro–Wilk test for normality distribution to ensure suitability for the statistical tests employed, considering significance at a p-value of 0.05 or lower. Descriptive statistics, including frequencies (percentages) and mean ± standard deviations (SD), were utilized to summarize the participants’ value general characteristics and the scores obtained on various scales. Parametric inferential statistics such as t-tests (ANOVA) and regression analyses were used to examine the differences and similarities between study variables. Pearson analysis was used to investigate the correlations found. A probability (p-) less than 0.05 was considered significant, and less than 0.001 was considered highly important). Finally, the PROCESS macro technique for SPSS was used to examine the mediation role of hardiness.

## Result

Results of our study Show that the mean score of the participant’s students was 20.02 ± 2.12; 55% of them were male, and 50.6% of them studied at the Nursing Technical Institute. Regarding the academic level, 64.4% of them studied at the first academic level, 56% of them were from urban areas, 96.1% of them were single, 67.9% had enough monthly income, and 22.8% of them were from upper Egypt. Concerning previous grades, 40.8% of them had excellent previous grades, and 77.1% of them had never used non-prescribed sedatives during exams. See Table 1.

**Table 1:** Demographic and academic characteristics of participants (*n* = 1932)

Personal data	Categories Mean ± SD	Mean ± SD/*N*(%)
Age		20.02 **±** 2.12
Gender	Male	1070 (55.4)
	Female	862 (44.6)
Marital statutes	Single	1855 (96.1)
	Married	77 (3.9)
Geographic area	Upper Egypt	440 (22.8)
	Eastern Egypt	293 (15.2)
	Western Egypt	357 (18.5)
	Delta area of Egypt	432 (22.3)
	The Center area of Egypt	410 (21.2)
Monthly income	Enough	1311 (67.9)
	Not enough	621 (32.1)
Residence	Rural	851 (44.0)
	Urban	1081 (56.0)
Academic level	First	1244 (64.4)
	Second	230 (11.9)
	Third	198 (10.2)
	Fourth	204 (10.6)
	Nursing interns	56 (2.9)
Previous grade	Excellent	641 (33.2)
	Very good	789 (40.8)
	Good	323 (16.7)
	Pass	81 (4.2)
	Fail	98 (5.1)
Intake sedatives during exams	Never	1489 (77.1)
	Sometimes	306 (15.8)
	Always	96 (5.0)
	Often	41 (2.1)

F: Anova test, t: T-test, P: P-value

Table 2 shows that nearly half, 49.7% of the participants, had high-level feedback sensitivity, 52.1% had a moderate level of hardiness, and 52.9% had a high level of test anxiety. The table also shows that there was a statistically positive correlation between feedback sensitivity and test anxiety at (*P* < 0.001). At the same time, there was a statistically significant negative correlation between hardiness feedback sensitivity and test anxiety at (*P* < 0.001).

**Table 2:** Descriptive analysis and correlation between studied variables (*n* = 1932)

Variable	1	2	3
Feedback sensitivity	1.00		
Hardiness	− 0.476^**^ (< 0.001)	1.00	
Test anxiety	0.942^**^(< 0.001)	− 0.682^**^(< 0.001)	1.00
Mean ± SD	119.5952 **±** 26.70433	41.1128 **±** 10.45850	35.9073 **±** 10.94283
High	(960) 49.7%	(114) 5.9%	(1022) 52.9%
Moderate	(462) 23.9%	(1007) 52.1%	(672) 34.8
Low	(510) 26.4%	(811) 42%	(238) 12.3%

r = Pearson Correlation, **Correlation is highly significant at the 0.01 level (2-tailed), α =

M, Mean; SD, Standard Deviation

Study results Clarify that the predictors of the participant’s students test anxiety according to liner regression model were monthly income at (B = 0.888, t = 0.1.764, *P* < 0.01), previous grade (B = 0.863, t = 3.314, *P* < 0.001), previous fail (B = 0.692, t = 2.957, *P* < 0.001), hardiness (B = 0.069, t = 2.160, *P* < 0.01) and feedback sensitivity (B = 144, t = 16.224, *P* < 0.001). See Table 3.

**Table 3:** Factors predicting test anxiety among participants using multiple linear regression analysis (*n* = 1932)

Model	B	SE	Beta	t	Sig.	CI	
						Lower bound	Upper bound
(Constant)	15.577	3.419		4.556	**< 0.001** ^**^	8.872	22.283
Age	0.017	0.158	0.003	0.106	0.915	0.294	0.327
Gender	0.741	0.487	0.111	1.52	0.125	-0.214	1.696
Education Level	0.059	0.049	0.038	1.20	0.230	0.037	0.155
Academic level	0.178	0.275	0.719	0.647	0.518	0.361	0.717
Residence	0.684	0.473	0.031	1.447	0.149	0.243	1.611
Marital statutes	0.134	1.350	0.002	0.100	0.921	2.512	2.781
Monthly Income	0.888	0.304	0.078	2.921	**< 0.01** ^*^	0.292	1.484
Previous Grade	0.863	0.260	0.083	3.314	< 0.01 ^**^	0.352	1.374
Pervious fail	0.892	0.212	0.126	2.957	< 0.01^**^	0.412	1.596
Intake sedatives during exams	0.491	0.464	0.490	1.058	0.291	0.401	0.581
Hardiness	− 0.069	0.032	− 0.065	-2.160	< 0.05	0.418	1.400
Feedback sensitivity	0.144	0.009	0.350	16.224	< 0.01	0.126	0.161
R2	0.423						
Adjusted R2	0.174						
F-test (p value)	34.407 < 0.001						

SE = Std. Error, CI = 95% Confidence Interval

Table 4 shows that feedback sensitivity directly affected test anxiety at (B = 0.1472, t = 16.8471, *P* < 0.001). Also, there was a statistically significant effect of feedback sensitivity on hardiness at (B=.-0.0878, t=-10.1028, *P* < 0.001) and hardiness on test anxiety at (B=-0.1377, t=-6.172, *P* < 0.001). Concerning the indirect effect, hardiness had a mediation role in the relationship between feedback sensitivity and test anxiety at (B = 0.126, t = 7.734, *P* < 0.001)—Table 4.

**Table 4:** Mediating effect of hardiness between feedback sensitivity and test anxiety (*n* = 1932)

**Direct effect**	**(B)**	**SE**	**t**	***p***	**LLCI**	**ULCI**
Feedback sensitivity → test anxiety	0.1472	0.0087	16.8471	< 0.001	0.1300	0.1643
Feedback sensitivity → Hardiness	− 0.0878	0.087	-10.1028	< 0.001	− 0.1048	− 0.0707
Hardiness → test anxiety	− 0.1377	0.0223	-6.172	< 0.001	− 0.1814	− 0.0939
**Indirect effect**				< 0.001		
Feedback sensitivity → Hardiness → test anxiety	0.126	0.0025	7.734	< 0.001	0.121	0.131
Total effect				< 0.001		
Feedback sensitivity → test anxiety	0.1592	0.0086	18.529	< 0.001	0.1424	0.1761

## Discussion

Students’ mental health and psychological resilience are critical for their academic success and professional competence in the demanding nursing field. However, several psychological factors can adversely affect their performance and well-being. Notably, hardiness, feedback sensitivity, and test anxiety are significant issues that warrant closer examination. By understanding the interplay between feedback sensitivity, hardiness, and test anxiety and addressing these psychological factors, educational institutions can enhance student well-being, improve academic outcomes, and better prepare students for the challenges of the nursing profession.

### Teat anxiety level among nursing students

Nursing students exhibit moderate to high characteristics of test anxiety in the current study; this matched with Khaira et al., 2023, who found that 62.5% of nursing students, 25.4%, experienced moderate test anxiety, and 2.1% experienced severe test anxiety. In addition, Dawood, 2016 and Qutishat et al., 2018 found considerable test anxiety among nursing students [45, 46].

These factors are inherent to their rigorous academic and clinical training. The high-stakes nature of nursing exams, which often determine progression in their programs and future licensure, creates immense pressure to perform well. This pressure is exacerbated by the fear of failure, which can have significant personal and professional consequences. Dreher and colleagues, 2019 documented rigorous academic standards and progression policies implemented by nursing programs. These programs must ensure that students possess the necessary knowledge and competencies to practice nursing safely [47]. French and colleagues’ 2023 scoping review of the literature indicates that empirical evidence does not support the heavy reliance on high-stakes final examinations in many university subjects. Traditional examinations, characterized as closed-book, individual, invigilated, time-constrained, summative, final, and high-stakes, have been found to possess limited pedagogical value [48].

Additionally, nursing students frequently face time constraints and high workloads, which can limit their preparation time and increase stress levels. The demanding clinical placements and the need to balance academic responsibilities with hands-on training further contribute to their anxiety [49]. Nursing students must manage substantial workloads, adhere to strict time constraints, participate in clinical placements, and meet high academic expectations throughout their education. Research has consistently demonstrated that nursing students experience higher stress levels than their peers in other disciplines [50, 51].

### Association between test anxiety and feedback sensitivity

Higher levels of feedback sensitivity are strongly associated with higher levels of test anxiety among current subjects. In the same line, Tobias & Ito, 2021 found that higher anxiety was linked to more excellent reactions to both negative feedback and mistakes as the task continued. This suggests that anxiety causes reactions to negative cues to be more persistent [52].

According to Lazarus and Folkman’s Cognitive Appraisal Theory (1984), individuals assess and interpret stressors in their environment, which affects their emotional responses. Feedback sensitivity may cause individuals to perceive feedback as more threatening or critical, thereby increasing anxiety, especially in evaluative situations like tests. Jones et al., 2021 [52] documented that those non-anxious individuals showed the opposite pattern, demonstrating better learning from positive feedback and a preference for it. They suggest that the difficulties with feedback-based learning in anxious individuals are due to changes in the mesolimbic dopaminergic system.

In addition, high feedback sensitivity can amplify the fear of failure. When students receive criticism, there is a well-established practice of referring to feedback that negatively evaluates someone’s work, performance, or qualities as “failure feedback.” [53, 54] Students often interpret such criticism as a sign of failure. These individuals might be overly concerned about making mistakes and receiving negative feedback, which can elevate anxiety levels during exams and other evaluative scenarios [55].

Individuals sensitive to feedback might engage in cognitive distortions, such as catastrophizing or overgeneralizing. Putwain et al. (2010) documented those cognitive distortions act as mediators with the test anxiety–-examination performance. They may view a single piece of negative feedback as indicative of their overall abilities, leading to increased test anxiety. Test anxiety is a fitting area to explore the impact of cognitive distortions, given its strong association with concerns about performance and failure, which are core characteristics of test anxiety. Moreover, test anxiety has been consistently linked with poorer performance in examinations [56].

### Test anxiety and hardiness among nursing students

The current students revealed a negative correlation between hardiness and test anxiety, which almost matched [57, 58] applied readiness training to students, effectively reducing test anxiety among students.

Hardy individuals are more resilient and adaptable. They view challenges as opportunities for growth rather than threats, which reduces the anxiety typically associated with tests [59]. Resilient individuals often have a growth mindset, believing abilities and intelligence can be developed with effort and practice. This mindset encourages them to embrace challenges, persist despite setbacks, and learn from criticism [60]. The belief that they can improve and succeed through hard work reduces the anxiety associated with tests.

In addition, individuals rarely adopt a problem-solving approach. This helps them break down the tasks required for exam preparation into manageable steps, develop effective study plans, and seek help when needed [61]. This proactive approach reduces the uncertainty and lack of control that often contribute to test anxiety.

### Feedback sensitivity and hardiness among nursing students

The negative correlation between feedback sensitivity and hardiness is almost matched by Karbakhsh et al. [62] who found that hardiness is associated with sensitivity in physicians. Students with high feedback sensitivity may perceive feedback, especially negative feedback, as personal criticism rather than constructive input [60], leading to feelings of helplessness and reducing their sense of control and commitment, which are critical components of hardiness [63].

High feedback sensitivity can also trigger a more robust stress response to feedback situations, making it more difficult for individuals to maintain a resilient and hardy attitude [64]. Stress can diminish one’s ability to view challenges as opportunities for growth, a critical aspect of hardiness [65]. Cognitive appraisal is another factor, as feedback-sensitive individuals may interpret feedback to emphasize personal shortcomings rather than areas for improvement, leading to alienation and a diminished sense of commitment [66]. Lastly, previous negative experiences with feedback can make individuals more sensitive to it in the future, further affecting their overall hardiness.

### Mediating effect of hardiness between feedback sensitivity and test anxiety

Our result showed that hardiness negatively influences test anxiety and feedback sensitivity, underscoring its role as a protective factor against anxiety in testing contexts. Similarly, Abdollahi et al., 2018 highlight the role of hardiness between perfectionism and test anxiety among students [67]. These results contribute to our adding to the body of literature by elucidating mechanisms that clarify the impact of perfectionism on test anxiety. The direct positive effect of feedback sensitivity on test anxiety suggests that individuals who are more attuned to and affected by feedback may experience heightened anxiety during tests. This sensitivity could amplify perceived stakes and pressure, increasing emotional distress. Conversely, the negative direct effect of feedback sensitivity on hardiness implies that those highly sensitive to feedback may struggle with developing resilience and adaptive coping mechanisms. This lack of hardiness could exacerbate anxiety responses by reducing one’s ability to manage stressors inherent in test-taking situations effectively.

According to Biggs and colleagues, 2024 hardiness is fundamental in maximizing psychological endurance and determining how long individuals can sustain effort. Self-control manages energy allocation, enhancing the capacity needed for endurance, while resilience is a psychological recharge mechanism. These factors are crucially influenced by environmental, psychological, and physiological stressors, which deplete the psychological battery [68].

Furthermore, the negative direct effect of hardiness on test anxiety indicates that individuals with lower levels of hardiness may be more susceptible to experiencing heightened anxiety during tests. Hardiness enhances one’s capacity to withstand stress and adversity. The indirect effect underscores the role of hardiness as a mediator between feedback sensitivity and test anxiety. This pathway suggests that feedback sensitivity influences test anxiety partially through its impact on hardiness. Individuals susceptible to feedback may perceive critiques or evaluations more negatively, which could erode their resilience over time.

In addition, our findings suggested that higher levels of feedback sensitivity, lower grades, previous academic failures, and lower hardiness scores are linked to increased test anxiety. The literature findings found academic standing played a significant role, with some studies indicating that senior students experienced higher levels of test anxiety [69, 70]. In contrast, others found that junior students faced more significant test anxiety [71]. The type of housing accommodations was also identified as a further demographic factor affecting Test Anxiety [72, 73]. However, our study found that residence did not show significant associations with test anxiety in this sample.

### Strengths and limitation

A notable strength is the multi-site design, which enhances the generalizability of the results by including a diverse sample of nursing students from various educational settings. Additionally, the use of linear regression and mediation analysis provides robust insights into the direct and indirect effects of feedback sensitivity and hardiness on test anxiety, offering practical implications for educational interventions.

Although the multi-site approach improves generalizability, the lack of randomization means that the findings may still be context-specific to the participating institutions, which could limit their applicability to other settings. Moreover, excluding participants with comorbid anxiety disorders may affect the findings’ relevance to students with more complex psychological conditions.

### Implications

The findings of this study underscore the need for educational strategies that effectively address both feedback sensitivity and hardiness to manage test anxiety among nursing students. Academic institutions should consider implementing targeted interventions that help students develop resilience and coping skills, enhancing their hardiness. Workshops or programs focused on stress management, effective feedback handling, and psychological resilience could be integrated into nursing curricula to support students in managing test anxiety more effectively.

Future research should explore the effectiveness of these interventions in diverse educational settings and among various student populations. Longitudinal studies could provide insights into how hardiness and feedback sensitivity improvements impact test anxiety over time. Additionally, research could examine other potential mediators and moderators in the relationship between feedback sensitivity and test anxiety, such as self-efficacy or social support. Investigating these aspects could lead to a more comprehensive understanding of the factors influencing test anxiety and inform the development of more effective, tailored interventions.

## Conclusion

Collectively, this research underscores the critical role of feedback sensitivity and hardiness in shaping test anxiety among nursing students. The findings reveal that while high feedback sensitivity correlates with increased test anxiety, enhanced hardiness serves as a mitigating factor. This suggests that educational interventions aimed at building hardiness and effectively managing feedback sensitivity could significantly reduce test anxiety.

## Data Availability

The corresponding author can provide the datasets used and/or analyzed for this study upon reasonable request.

## Abbreviations

IFOS: Instructional Feedback Orientation Scale
TAQ: Test Anxiety Questionnaire
